# Preclinical Activity of ARQ 087, a Novel Inhibitor Targeting FGFR Dysregulation

**DOI:** 10.1371/journal.pone.0162594

**Published:** 2016-09-14

**Authors:** Terence G. Hall, Yi Yu, Sudharshan Eathiraj, Yunxia Wang, Ronald E. Savage, Jean-Marc Lapierre, Brian Schwartz, Giovanni Abbadessa

**Affiliations:** ArQule, Inc., Burlington, MA, United States of America; Institute of Bioengineering and Nanotechnology, SINGAPORE

## Abstract

Dysregulation of Fibroblast Growth Factor Receptor (FGFR) signaling through amplifications, mutations, and gene fusions has been implicated in a broad array of cancers (e.g. liver, gastric, ovarian, endometrial, and bladder). ARQ 087 is a novel, ATP competitive, small molecule, multi-kinase inhibitor with potent *in vitro* and *in vivo* activity against FGFR addicted cell lines and tumors. Biochemically, ARQ 087 exhibited IC_50_ values of 1.8 nM for FGFR2, and 4.5 nM for FGFR1 and 3. In cells, inhibition of FGFR2 auto-phosphorylation and other proteins downstream in the FGFR pathway (FRS2α, AKT, ERK) was evident by the response to ARQ 087 treatment. Cell proliferation studies demonstrated ARQ 087 has anti-proliferative activity in cell lines driven by FGFR dysregulation, including amplifications, fusions, and mutations. Cell cycle studies in cell lines with high levels of FGFR2 protein showed a positive relationship between ARQ 087 induced G1 cell cycle arrest and subsequent induction of apoptosis. In addition, ARQ 087 was effective at inhibiting tumor growth *in vivo* in FGFR2 altered, SNU-16 and NCI-H716, xenograft tumor models with gene amplifications and fusions. ARQ 087 is currently being studied in a phase 1/2 clinical trial that includes a sub cohort for intrahepatic cholangiocarcinoma patients with confirmed FGFR2 gene fusions (NCT01752920).

## Introduction

The FGFR family of tyrosine kinase receptors (FGFR1-4), and their ligands, the fibroblast growth factors (FGFs), play an important role in multiple signal transduction pathways including mitogen-activated protein kinases (MAPK), phosphatidylinositol 3-kinase (PI3K) phospholipase Cγ (PLCγ), protein kinase C (PKC), and signal transducers and activator of transcription (STAT). FGFR activation leads to a series of cellular signaling events including increased cellular proliferation, differentiation, and migration [1, 2].

Dysregulation in the FGFR tyrosine kinase family has been implicated in a number of human cancers, including cholangiocarcinoma, squamous non-small cell lung cancer (sqNSCLC), small cell lung cancer (SCLC), gastric, breast, ovarian, endometrial, and bladder carcinomas [1, 3–5]. In human cancers, FGFRs have been found to be dysregulated by multiple mechanisms, including aberrant expression, mutations, gene fusions, and amplifications [6–9]. Such genetic alterations have been implicated in the oncogenicity of several tumor models, suggesting that therapeutic targeting of FGFRs may benefit cancer patients. Inhibition of FGFR *in vitro* and *in vivo* has been shown to reduce proliferation of FGFR-dependent cancer cells and induces cell death [3, 4, 8, 10–12]. Currently there are a number of therapeutic agents in clinical development that either specifically target FGFRs, or target them as part of a spectrum of kinases [13–18].

Recently there has been an increased interest in the oncogenic potential of FGFR gene fusions in a number of cancer types including, lung, blood, brain, breast, prostate, and biliary tract. [16, 19–25]. Examples of FGFR fusion driven cancers include: 8p11 myeloproliferative syndrome, a rare stem cell disorder, which contains a number of FGFR1 fusions including FGFR1OP-FGFR1 [16], and glioblastoma multiforme with FGFR3-TACC3 fusions which are found in 3–7% of all GBM [26].

One tumor type where gene fusions appear to be particularly common (15–45%) is the intrahepatic form of cholangiocarcinoma (iCCA) [5, 20, 21, 27–32]. Cholangiocarcinoma is an epithelial malignancy of biliary tract that is subcategorized based on its anatomic location within the biliary tree, with the intrahepatic form arising from the intrahepatic biliary ductal system [33]. A large number of FGFR2 gene fusions have been identified in cholangiocarcinoma, and FGFR inhibitors have shown to be partially effective in reducing tumor burden in patients [5, 20, 21, 27, 28]. Currently, there have been over 11 fusion partners for FGFR2 identified in intrahepatic cholangiocarcinoma patient samples S1 Table. Of note, FGFR2-AHCYL, FGFR2-KIAA1598, and FGFR-PPHLN1 are novel gene fusions that have not been observed in other cancer types [5, 20, 27, 34].

We report data on the pharmacological inhibitory functions of ARQ 087, a small-molecule kinase inhibitor, with potent activity against the FGFR family. ARQ 087 demonstrated inhibitory activity against FGFR2 amplifications and gene fusions *in vitro* and *in vivo*. In multiple xenograft models, ARQ 087 effectively inhibited the growth of FGFR2 driven tumors.

## Materials and Methods

### Cell lines and reagents

Recombinant and FGFR2 kinase domains in the unphosphorylated state were generated as reported previously [35]. The A2780, AN3CA, COS-1, J82, K-562, KATO-III, KG-1, NCI-H716, RT4, SKOV-3, SNU-16, and SW-780 cells used in this study were obtained from ATCC (Manassas, VA). The MFE-280, MFE-296, MFM-223, and RT-112 cells were acquired from the Deutsche Sammlung von Mikroorganismen und Zellkulturen GmbH (Berlin, Germany). All cell lines were maintained in either RMPI 1640 or DMEM in the presence of 10% FBS in a 37°C humidified incubator with 5% CO_2_ and were passaged for fewer than 6 months after being received. Re-authentication was not performed. High capacity RNA-to-DNA kit and master mix for real time qPCR analysis were purchased from Life Technologies (Grand Island, NY). The primary antibodies for Western blot analysis were purchased from Cell Signaling Technology (Danvers, MA), R&D Systems (Minneapolis, MN), Santa Cruz Biotechnology (Dallas, TX), and β-actin Sigma-Aldrich (St. Louis, MO). ARQ 087 was supplied by ArQule, Inc.

### Determination of K_i_ and mode of inhibition

Kinase inhibitory activity of ARQ 087 was determined for the recombinant FGFR1 (Cat#08133, Carna BioScience, Kobe, Japan) or FGFR2 proteins (Cat#08134, Carna BioScience, Kobe, Japan), utilizing a biotinylated PYK2 peptide substrate (biotin-AGAGSIESDIYAEIPDETC-NH2, Biopeptide, San Diego, CA), and ATP (Cat#12215226, Roche, Indianapolis, IN), with AlphaScreen^™^ technology (PerkinElmer, Waltham, MA).

ARQ 087 was titrated in DMSO utilizing a 3-fold dilution scheme, and then diluted 10-fold further in deionized water for a final DMSO concentration of 10%. A volume (2.5 μL) of these dilutions or vehicle was added to each well of a reaction plate (Cat#3642, Corning life sciences, Corning, NY). FGFR1 or FGFR2 was added to assay buffer (50 mM Tris, pH 8.0, 0.02 mg/mL BSA, 10 mM MgCl_2_, 1 mM EGTA, 10% glycerol, 0.1 mM Na_3_PO_4_, 1 mM DTT) to each well in a volume of 17.5 μL for a final concentration of 0.50 or 0.25 nM, respectively. After a 30-minute pre-incubation period, ATP and substrate were added in assay buffer (5 μL) for final concentrations of 0–1,000 μM ATP and 80 nM biotinylated-PYK2, for a final reaction volume of 25 μL. The plates were incubated for 60 minutes at room temperature, and then stopped in the dark by the addition of 10 μL stop/detection mixture prepared in assay buffer containing EDTA, AlphaScreen^™^ Streptavidin Donor and P-TYR-100 Acceptor beads for final concentrations of 10 mM EDTA and 500 ng/well of both AlphaScreen^™^ Donor and Acceptor beads. Assay plates were incubated for 60 minutes at room temperature in the dark, and the plates were read on a Perkin Elmer (Waltham, MA) Envision Multilabel plate reader (excitation wavelength: 640 nm, emission wavelength: 570 nm). The effect of enzyme concentration was applied for tight-binding inhibitors, and if necessary, the IC_50_ values were converted into K_i_ values if the enzyme concentration was above the IC_50_ values under the assay conditions utilized [36, 37]. The analysis was performed using the DynaFit software program (Biokin, Watertown, MA).

### Kinase auto-phosphorylation assays

FGFR kinase auto-activation activity was monitored using a continuous spectrophotometric assay as described previously [35]. In this assay, the consumption of ATP is coupled via the pyruvate kinase/lactate dehydrogenase enzyme pair to the oxidation of NADH, which is monitored through a decrease in absorption at 340 nm. The assay mixture contained 100 mM Tris (pH 8.0), 10 mM MgCl_2_, 1 mM phosphoenolpyruvate, 0.28 mM NADH, 89 U/mL pyruvate kinase, 124 U/mL lactate dehydrogenase and 2% DMSO. Reactions were initiated by the addition of 1 mM ATP to assay mixtures containing enzyme incubated with various concentrations of ARQ 087 and the decrease in absorbance was monitored at 30°C using a Safire II plate reader (Tecan Männedorf, Switzerland) Safire II plate reader. Unphosphorylated forms of FGFR1 or FGFR2 kinase domains were titrated with increasing concentrations of ARQ 087 and inhibition of kinase activation delay was measured.

### Kinase biochemical profiling

ARQ 087 was first profiled against 298 kinases at a concentration of 0.1 μM (Carna Biosciences, Kobe, Japan). Kinases that inhibited greater than 50% at 0.1 μM by ARQ 087 were subjected to IC_50_ determination in a subsequent study (Carna Biosciences).

### Cell proliferation assays

Cells were seeded at 3000–5000 cells per well with 130 μL media in 96-well tissue culture treated plates. The cells were incubated overnight and subsequently treated with 3-fold serial dilutions of ARQ 087 starting at 100 μM. The cells were returned to a 37°C humidified incubator for 72 hours. MTS Reagent (Promega, Madison, WI) was supplemented with a 1:20 dilution of 0.92mg/mL phenazine methosulfate (PMS, Sigma-Aldrich, St. Louis, MO). Thirty microliters of the MTS/PMS reagent were added to each well, and the plates were incubated at 37°C for an additional 4 hours. The absorbance was measured at 490 nM using the Victor^™^ or Envision^®^ microplate reader (Perkin Elmer, Waltham, MA). GI_50_ values were calculated using Activity Base and XLfit^™^ (IDBS, Surrey, United Kingdom). Anti-proliferative cell-based tyrosine kinase assays for FGFR1, FGFR2, FGFR3, FGFR4, FGFR2 fusions (AFF3, TEL, BICC1, CASP7, CCD6, CIT), FGFR3-BAIAP2L1, ARG, LCK, KIT, RET, SRC, FYN, FLT4 (VEGFR3), LYN, PDGFR-β, FLT1 (VGFR1), PDGFR-α, IGF1R, KDR (VEGFR2), FMS (CSF1-R) and EphA1 were conducted at Advance Cellular Dynamics (San Diego, CA).

### Exogenous expression of FGFRs in COS-1 cells

COS-1 cells were transfected with mammalian expression vectors encoding full-length *FGFR1*, *FGFR2*, *FGFR3* or *FGFR4* [OmicsLink AviTag ORF, M17 (GeneCopoeia, Rockville, MD) using Lipofectamine 2000 transfection reagent (Life Technologies). Forty-eight hour post-transfection, the media were removed and replaced with fresh culture media. Cells were pre-treated with various concentrations of ARQ 087 for 2 hours prior to being stimulated with 100 pM of a mixture of FGF1/FGF2/FGF7 for 15 minutes. Cells were lysed and subjected to Western blotting analysis as described below. The anti-AVI-tag antibody was used to detect expression of total FGFRs (GeneCopoeia).

### Cell cycle analysis

Cells were plated and incubated at 37°C overnight and subsequently treated with 0.1 μM or 1 μM of ARQ 087 for 24 or 72 hours. The cells were fixed and stained with Cycletest Plus Reagent kit (BD Biosciences, Franklin Lakes, NJ) according to the manufacturer’s instructions, and cell cycle profiles were analyzed using a FACS Calibur flow cytometer (BD Biosciences).

### Western blotting analysis

Cells were plated in 6-well plates with 1 mL growth media per well and incubated at 37°C overnight. The cells were treated with various concentrations of ARQ 087 for 2, 24, 48 or 72 hours followed by a 15-minute stimulation with or without 100 ng/mL of FGF1/FGF2/FGF7. Media were removed from the each well and replaced with 150 μL of 1X E-Page Loading buffer. Lysates were collected and transferred to 96-deep well plates and sonicated. Electrophoresis was performed on 4–12% Tris-Glycine gels (Life Technologies, Carlsbad, CA) or on E-PAGE^™^ gels (Life Technologies, Carlsbad, CA). Proteins were transferred to PVDF membranes (Life Technologies, Carlsbad, CA) using the BioRad Mini Trans-Blot^®^ system (BioRad, Hercules, CA) for 4–12% Tris-Glycine gels (Life Technologies, Carlsbad, CA), or by the iBlot^®^ system (Life Technologies, Carlsbad, CA) for E-PAGE^™^ gels (Life Technologies, Carlsbad, CA). Membranes were blocked with either 5% (w/v) BSA in 1X TBST or Odyssey blocking buffer (LI-COR, Lincoln, Nebraska) then probed with the appropriate primary antibodies overnight at 4°C. Secondary antibodies labeled with appropriate near-infrared dyes (LI-COR) or horseradish peroxidase (HRP, Santa Cruz Biotechnology, Dallas, TX) were then used. The membranes were either scanned using the Odyssey infrared scanner (LI-COR) or developed with ECL reagents (GE Healthcare, Little Chalfont, Buckinghamshire, UK) and imaged on the Fuji LAS 3000 system. When quantitation was feasible, the intensities of the bands were quantitated using the accompanying software, and EC_50_ values were determined.

### *In vivo* studies

All experimental procedures and surgical manipulations (if any) were approved in accordance with ArQule’s (Burlington, MA) Institutional Animal Care and Use Committee (IACUC), which included a licensed veterinary professional. During the conduct of the studies animal health was monitored, and animals that lost greater than 20% body weight, or were moribund were euthanized prior to the completion of the study. Tumor size was monitored regularly during all xenograft studies and animals whose tumor size exceeded the maximum allowable size (2000 mg or >20% of body weight), or interfered with the general health of the animal, were euthanized prior to the completion of the study. Animals were euthanized by CO_2_ inhalation. For the SNU-16 and NCI-H716 xenograft studies there were no unscheduled deaths. In the BaF3-FGFR2 model there were three unscheduled deaths; two animal handling errors, and one animal was sacrificed early for health reasons (unclear if this was drug related). Individual animal and tumor weights for all animals in this study are included with the supplemental information S1 Appendix.

Six week old female NCr *nu/nu* mice (for SNU-16) or CB-17 SCID female mice (for NCI-H716 and BaF3 models) were purchased from Taconic Farms (Germantown, NY) and allowed to acclimate for greater than 2 weeks. Mice were housed in sterile micro isolator cages with 5 mice per cage and were received food and water *ad libitum*. Tumor cells were suspended in 50% Matrigel (lot# A9618-BD, Bioscience) and sterile Hanks Balanced Salt Solution (HBSS) for the NCI-H716 model or in HBSS alone for SNU-16. Each mouse was implanted subcutaneously with 5x10^6^ cells (SNU-16) or 8x10^6^ cells (NCI-H716) or 2x10^6^ cells (BaF3) to the upper right flank area. Tumor measurements and body weights were collected two to three times per week with electronic calipers and balance. Tumor weight (mg) was calculated from the equation: length x (width)^2^/2. This formula was used to calculate tumor volume assuming unit density 1 mg = 1 mm^3^. Treatment was initiated when tumor burden was between 100–200 mgs. Percent inhibition or tumor growth inhibition (TGI) was calculated using the following formula: 100-[mean tumor value of treated / mean tumor value of control] x 100.

For in vivo pathway inhibition studies, female NCr *nu/nu* mice (SNU-16) or CB17 SCID mice (NCI-H716) with well established (~400 mg) subcutaneous tumors were given a single oral dose of ARQ 087 or vehicle control. Plasma and tumor samples were collected 4 hours post single dose. For all *in vivo* experiments, ARQ 087 was formulated in DMA: cremophor EL: propylene glycol: 0.2 M acetate buffer, pH 5 (10:10:30:50) and administered orally. Dosing volume for all groups was 10 mL/kg or 0.1 mL/10 g body weight.

### Immunohistochemistry

Xenograft tumor tissues were collected after the 4-hour drug treatment, fixed for 16–24 hours in 10% neutral buffered formalin (NBF) and embedded in paraffin. Immunohistochemistry (IHC) was performed on the tumor tissue sections (5 μm thickness) for a panel of biomarkers. After deparaffination and rehydration, antigen retrieval was conducted in a decloaking chamber for 30 minutes at 95°C, followed by 10 seconds at 90°C using a 1 μM EDTA solution pH 8.0 (Cat# S2505, Poly Scientific, Bay Shore, NY) for total FGFR2, phospho-FGFR, and phospho-FRS2α; 10 mM Citrate buffer pH 6.0 (Cat# S2506, Poly Scientific, Bay Shore, NY) for phospho-ERK. IHC was performed using an autostainer 480 (Lab vision UK Ltd). These antibodies were diluted in 1% BSA/TBST. The sections were incubated with primary antibody for 30 minutes and then with secondary antibody (or the polymer) for 25 minutes at ambient temperature. Rabbit on Rodent HRP-Polymer (Cat# RMR622, Biocare Medical, Concord, CA) or Goat anti-rabbit HRP (Cat# 111-035-144, Jackson ImmunoResearch Laboratories, West Grove, PA) was used as a secondary antibody. 3,3’-diaminobenzidine (DAB) was used as the chromogen and the slides were counter-stained with hematoxylin after IHC.

### Statistical Analysis

For *in vivo* studies, all statistical analyses were performed using an unpaired *t* test. Data were presented as mean ± SEM. A *p* value of less than 0.05 was considered to be significant.

## Results

### Biochemical and kinetic data for ARQ 087

#### Kinase inhibition, selectivity, and mode of inhibition

**Fig 1:**
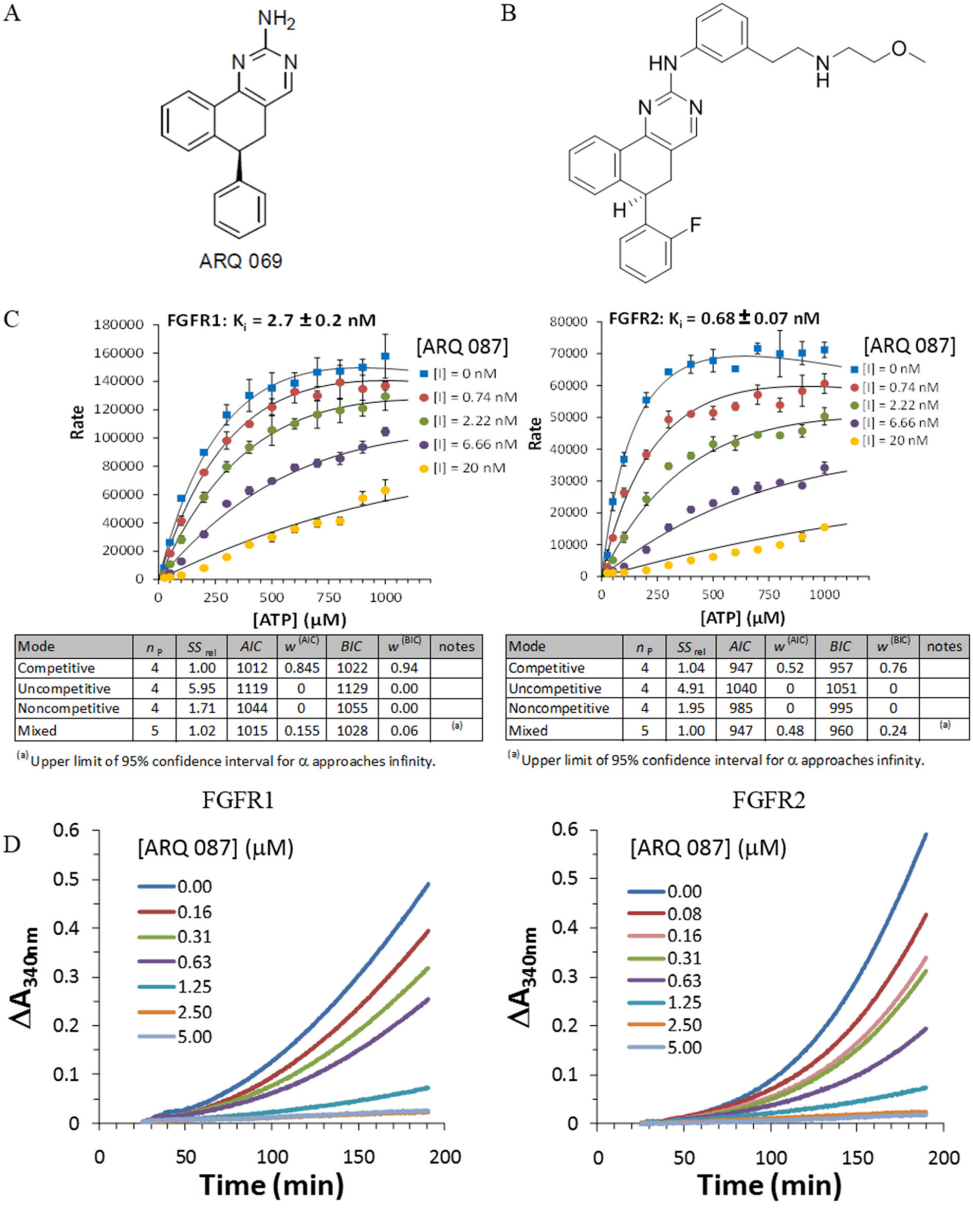
Mode of FGFR inhibition for ARQ 087. (A) ARQ 069. (B) ARQ 087. (C) Enzyme kinetic analysis was performed to determine the mode of inhibition of ARQ 087 with FGFR1 and FGFR2. Concentrations of ATP [ATP] and ARQ 087 [I] are indicated on the graphs. The rate plotted is the AlphaScreen^™^ signal obtained from the plate reader with background correction. The experiments were conducted in triplicates and the means and the standard deviations were plotted. The number of parameter (*n*_p_), the sum of squares rel (*SS*_rel_), Akaike information criterion (*AIC*), weight of AIC (*w*^(AIC)^), Bayesian information criterion (*BIC*) and the weight of BIC (*w*^(BIC)^) for each binding mode were determined by the DynaFit software and are summarized below the plots. The dissociation constant (*K*i) of ARQ 087 for FGFR1 and FGFR2 are shown. (D) The effect of ARQ 087 on the activation of FGFR1 and FGFR2 was examined in a continuous autophosphorylation assay.

We previously reported the *in silico* design and characterization of 5, 6 dihydrobenzo[*h*]quinazolin-2-amine as a novel chemical series of FGFR kinase inhibitors [35]. ARQ 069 Fig 1A, containing this core structure, was shown to bind the inactive conformation of FGFR1. The crystal structure of the FGFR1/ARQ 069 complex indicated that the aminopyrimidine moiety of the core participates in a hinge interaction and the hydrophobic region of the core stabilizes the downward G-loop conformation through non-polar interactions. ARQ 087 Fig 1B is an ATP competitive analogue of ARQ 069, which has been optimized for cellular potency and drug-like properties.

ARQ 087 is prepared as follows [38]: from (*R*)-4-(2-fluorophenyl)-3,4-dihydronaphthalen-1(2*H*)-one, using DMF-DMA overnight at 100°C, the corresponding enaminone was prepared. The (*R*,*E*)-2-((dimethylamino)methylene)-4-(2-fluorophenyl)-3,4-dihydronaphthalen-1(2*H*)-one was reacted with 1-(3-(2-hydroxyethyl)phenyl)guanidine in ethanol under sodium ethoxide action, giving the corresponding substituted aminopyrimidine. The methane sulfonate was prepared under standard conditions, giving (*R*)-3-((6-(2-fluorophenyl)-5,6-dihydrobenzo[*h*]quinazolin-2-yl)amino)phenethyl methanesulfonate which was then subjected to the displacement of the the methanesulfonate by 2-methoxyethylamine in presence of trimethylamine, giving after acidic work up, (*R*)-6-(2-fluorophenyl)-*N*-(3-(2-((2-methoxyethyl)amino)ethyl)phenyl) -5,6-dihydrobenzo[*h*]quinazolin-2-amine (ARQ 087) as a HCl salt.

ARQ 087 inhibited wild-type FGFR1, FGFR2, and FGFR3 with biochemical IC_50_ values in the 1.8–4.5 nM range, and FGFR4 with somewhat lower potency Table 1. To understand the mechanism by which ARQ 087 inhibited FGFRs, kinetic experiments were performed with ARQ 087 using FGFR1 and FGFR2 at a range of ATP concentrations, and the data were analyzed using the DynaFit software program. Michaelis-Menten graphs were generated Fig 1C, and four inhibition modes (competitive, uncompetitive, noncompetitive, and mixed) were evaluated using both the Akaike information criterion (*AIC*) and the Bayesian information criterion (*BIC*) to determine the best fitting model. The ATP competitive model produced the lowest *AIC* and *BIC* values, indicating that ARQ 087 is a competitive inhibitor of both FGFR1 and FGFR2 Fig 1C. Additionally, from the Michaelis-Menten plots, the *K*_i_ values of FGFR1 and FGFR2 were determined to be 2.7 ± 0.2 nM and 0.68 ± 0.07 nM, respectively, demonstrating a strong inhibitory potency of ARQ 087 for FGFR1 and FGFR2 Fig 1C.

**Table 1:** ARQ 087 biochemical activity.

Kinase	IC_50_ (nM)	Kinase	IC_50_ (nM)
FGFR2	1.8	PDGFRα	9.5
FGFR1	4.5	QIK	9.7
FGFR3	4.5	VEGFR1	11
FGFR4	34	SRC	11
RET	3	ABL	14
DDR2	3.6	EPHA1	15
FMS	3.8	CSK	17
PDGFRβ	4.1	FGR	17
LCK	6.2	LYN	17
YES	7.6	VEGFR2	21
ARG	7.9	VEGFR3	31
KIT	8.2	IGFR	>100

The biochemical IC_50_ values of ARQ 092 against 298 kinases were determined (Carna Biosciences)

Activation of FGFR kinases requires auto-phosphorylation on multiple tyrosine residues [39, 40]. Inhibition of this auto-activation reaction by ARQ 087 was evaluated by titrating increasing concentrations of ARQ 087 with unphosphorylated form of FGFR1 and FGFR2 kinases in an auto-phosphorylation assay. ARQ 087 inhibited the auto-phosphorylation of FGFR1 and FGFR2 in a dose-dependent manner Fig 1D. This observation suggests that ARQ 087 targets the un-phosphorylated or inactive form of the kinase in addition to inhibiting the active form, a hallmark of Type I inhibitors targeting the kinase by ATP competitive mechanism [41].

#### Biochemical activity

The selectivity of ARQ 087 across the kinome, was evaluated against a panel of 298 kinases at a concentration of 0.1 μM. Among 298 kinases assayed (kinase domain), approximately 50 (including FGFR1, FGFR2, FGFR3 and FGFR4) were inhibited by greater than 50% by ARQ 087. Examination of the IC_50_ values of this subset of kinases revealed that 18 wild-type kinases (excluding FGFRs), exhibited sensitivities to ARQ 087 within a 3- to 10-fold range of the IC_50_ value for FGFR2 Table 1. In biochemical assays, ARQ 087 appears to be most potent against FGFR1/2, however activity is also seen in a number of other kinases including RET, VEGFR, and KIT Table 1. The biochemical data suggest that ARQ 087 is a multi-kinase inhibitor that is highly potent against FGFR1/2/3.

#### In vitro results with ARQ 087 in cells with FGFR genetic alterations

**Fig 2:**
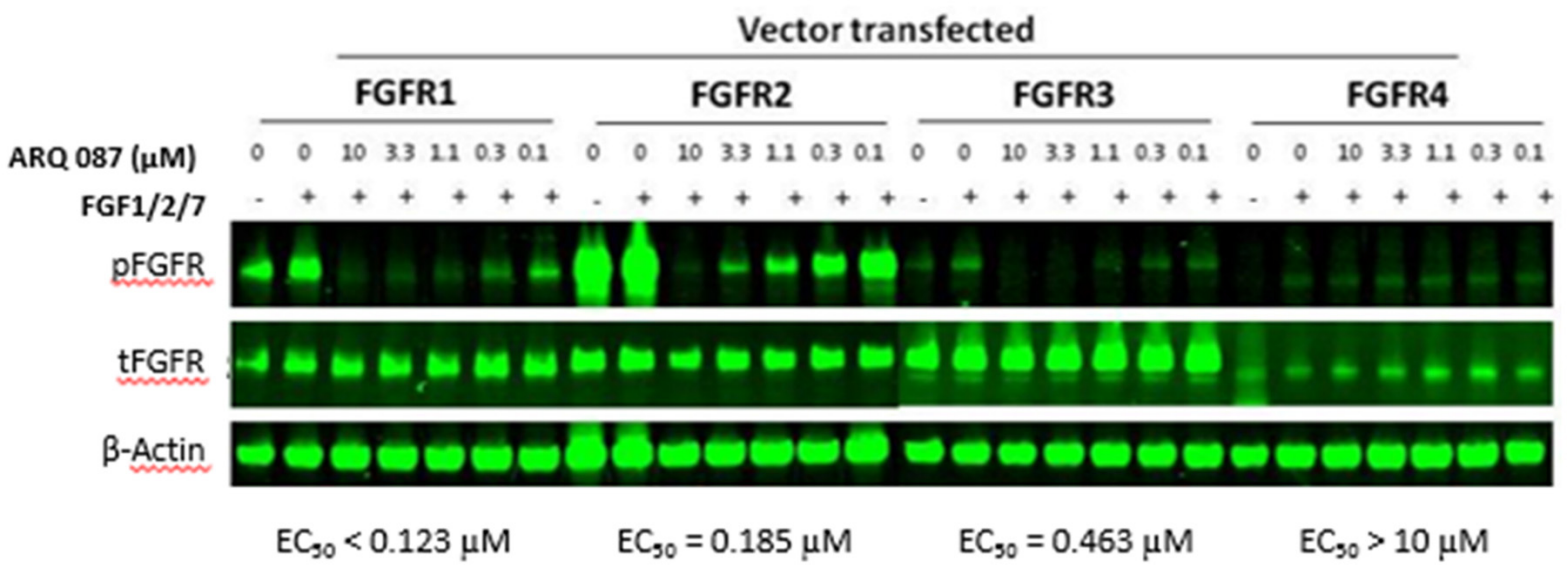
ARQ 087 inhibits FGFR phosphorylation. COS-1 cells ectopically expressing FGFR1, FGFR2, FGFR3 or FGFR4 were treated with the indicated concentrations of ARQ 087 for 2 hours followed by stimulation with 100 pM of FGF1/2/7 for 15 minutes. Total and phospho-FGFR was assessed by Western blot analyses. β-Actin was used as a loading control. The EC_50_ values of individual FGFR family members are shown.

Inhibition of FGFRs in transfected cell lines: To determine whether ARQ 087 inhibits the phosphorylation of FGFR1, FGFR2, FGFR3, and FGFR4 in cells, we over-expressed full-length FGFR1, FGFR2, FGFR3 and FGFR4 in COS-1 cells and examined the effect of ARQ 087 on phosphorylation of FGFRs (there is no antibody available that specifically recognizes the phosphorylation of each individual FGFR isoforms). We found that ARQ 087 inhibited the phosphorylation of FGFR1, FGFR2, FGFR3, and FGFR4 with EC_50_ values of < 0.123 μM, 0.185 μM, 0.463 μM, >10 μM respectively Fig 2.

We also examined the anti-proliferative effect of ARQ 087 in a panel of Ba/F3 cell lines that had been engineered to be dependent on individually over expressed tyrosine protein kinases for survival, including cell lines dependent on individual FGFR isoforms and FGFR fusions. The GI_50_ values for Ba/F3-FGFR1, Ba/F3-FGFR2, Ba/F3-FGFR3, and Ba/F3-FGFR4 ranged between 232 nM and 1346 nM, with Ba/F3-FGFR2 the most sensitive cell line to ARQ 087 followed by Ba/F3-FGFR1 and Ba/F3-FGFR3 Table 2. FGFR fusions were also examined in the Ba/F3 system, including TEL-FGFR2, FGFR2-AAF3, FGFR2-BICC1, FGFR2-CASP7, FGFR2-CCDC6, FGFR2-CCDC6, and FGFR3-BAIAP2L1. The GI_50_ values were between 39.9 nM and 1121 nM, with FGFR3-BAIAP2L1 as the most sensitive cell line Table 2.

**Table 2:** ARQ 087 GI_50_ in Ba/F3 transfected cell lines.

Kinase	GI_50_ (nM)	Kinase	GI_50_ (nM)
FGFR3-BAIAP2L1	34.9	FGFR2-CCD6	1121
FGFR2-CIT	39.5	FYN	1329
TEL-FGFR2	59.8	FGFR3	1337
FGFR2	232	FGFR4	1346
ARG	247	FLT4 (VEGFR3)	1354
LCK	287	LYN	1363
FGFR2-AFF3	337	PDFGR-β	1376
FGFR2-CASP7	349	FLT1 (VGFR1)	1407
FGFR1	355	PDFGFR-α	1567
KIT	474	IGF1R	1546
FGFR2-BICC1	990	KDR (VEGFR2)	1557
RET	991	FMS	1710
SRC	1091	EphA1	5381

In addition to FGFR-driven models, 15 other kinases that were potently inhibited by ARQ 087 biochemically were also investigated in the Ba/F3 engineered cell lines. These data reinforce the biochemical data that suggests ARQ 087 does inhibit kinases outside of the FGFR family in cellular assays. However, the majority of kinases had GI_50_ values above 1000 nM, with the exception of ARG, LCK, KIT, FGFR1-2, and FGFR fusions as shown in Table 2. These results demonstrate that ARQ 087 has the greatest potency in cell lines dependent on FGFR-fusions, followed by wild-type FGFR1/2 isoforms. ARQ 087 appears to hit a number of other kinases with equipotency to FGFR3/4.

#### Inhibition of FGFR pathway in cancer cell lines

**Fig 3:**
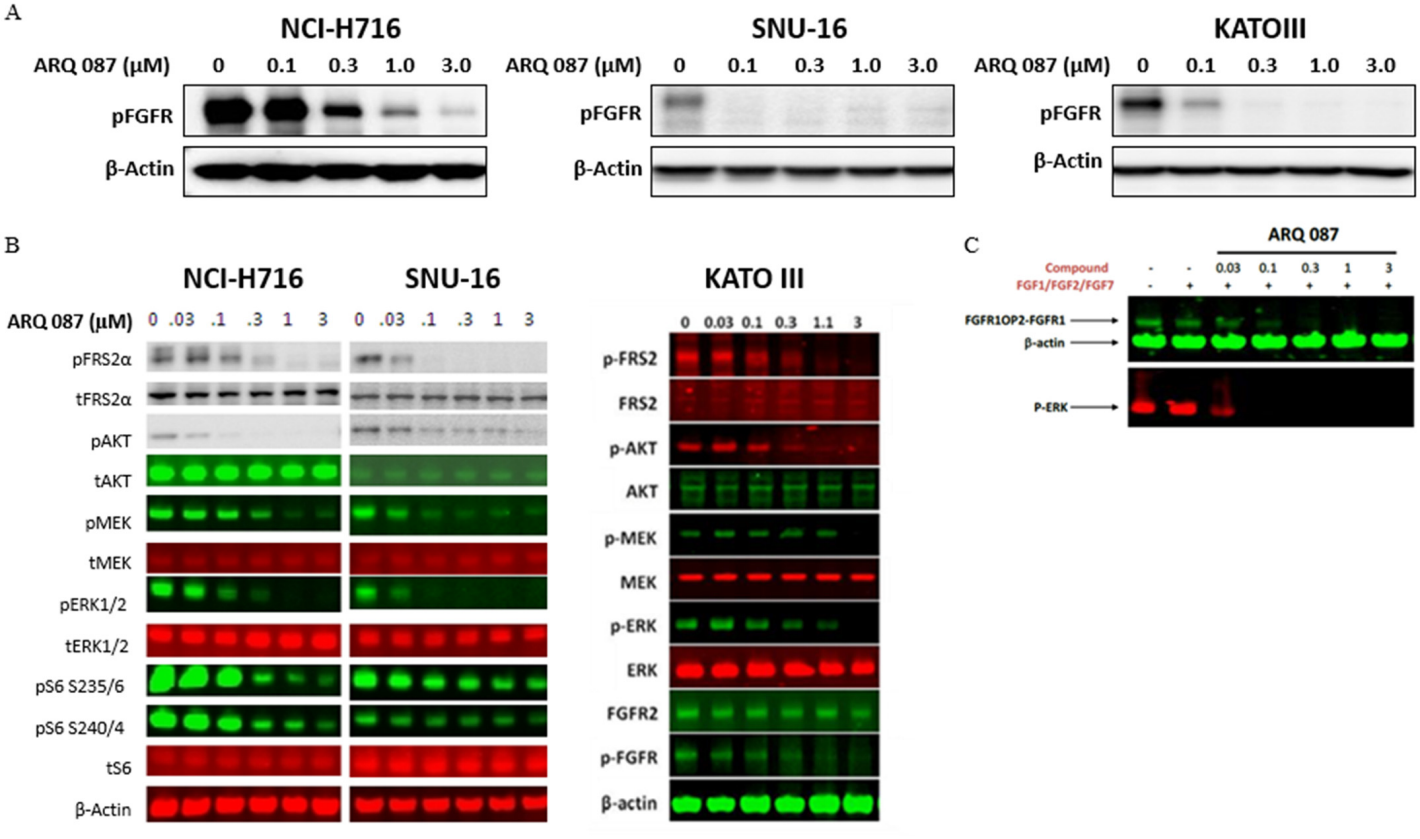
ARQ 087 inhibits the FGFR pathway in cancer cell lines. (A) NCI-H716, SNU-16, and KATO-III cells were treated with the indicated concentrations of ARQ 087 for 2 hours, and phospho-FGFR was assessed by Western blot analyses. β-Actin was used as a loading control. (B) NCI-H716 and SNU-16 cells were treated with increasing concentrations of ARQ 087 from 0 μM to 3 μM for 2 hours. The quantity of indicated protein was assessed by Western blot analyses. β-Actin was used as a loading control. (C) KG-1 cells were treated with indicated concentrations of ARQ 087 for 2 hours followed by stimulation with a mixture of FGF1/FGF2/FGF7 for 15 minutes. Cell lysates were analyzed by Western blot to determine the expression of phospho-FGFR, phospho-ERK, and β-actin.

FGFR pathway inhibition was examined in three solid tumor lines, the colorectal cancer cell line (NCI-H716), and gastric cancer cell lines (SNU-16 and KATO-III), all these cell lines are FGFR2 amplified [10, 11, 42, 43]. As shown in Fig 3A, ARQ 087 inhibited the phosphorylation of FGFRs in all three cell lines. Since all three of these cell lines express high levels of FGFR2 protein (NCI-H716, SNU-16, and KATO-III), we surmise that the pFGFR signal knockdown detected in Fig 3A was likely due to the inhibition of pFGFR2.

We next examined the signal transduction pathways activated downstream of FGFR in three FGFR2-dependent (NCI-H716, SNU-16, and KATO-III) cancer cell lines. ARQ 087 inhibited the phosphorylation of FGFR2 and its immediate downstream substrate, FRS2α, as well as the phosphorylation of further downstream components MEK, ERK and AKT Fig 3B. Based on these results, we conclude that ARQ 087 inhibits the FGFR signaling pathway in these FGFR2 over-expressing cancer cells.

In addition, we also tested ARQ 087 in the acute myeloid leukemia cell line KG-1. This cell line is driven FGFR1 fusion gene, FGFR1OP-FGFR1 [16]. Here we observed that ARQ 087 inhibits the phosphorylation of FGFR1OP-FGFR1 as well as the downstream marker, pERK Fig 3C. From this we conclude that ARQ 087 is able to inhibit the FGFR signaling pathway in this FGFR1 fusion driven cell line.

### Growth inhibition in FGFR dysregulated cell lines

We then evaluated the anti-proliferative effect of ARQ 087 in a number of cell lines that included cells with amplified FGFR, mutant FGFR, translocated FGFR, or unknown FGFR status. ARQ 087 demonstrated good potency in cell lines with FGFR2 amplifications and fusions. NCI-H716, SNU-16, and KG-1, were some of the most sensitive cell lines in this panel Table 3.

**Table 3:** ARQ 087 activity in FGFR dysregulated cell lines.

Cancer	Cell Line	FGFR Status [ref.]	ARQ 087 GI_50_ μM
Colon	NCI-H716	FGFR2 amp / FGFR2-COL14A1 fusion [44]/[45]	0.10
Gastric	SNU-16	FGFR2 amp / PDHX-FGFR2 fusion [11]/[45]	0.10
Blood	KG-1	FGFROP2-FGFR1 fusion [46]	0.13
Gastric	KATO-III	Amplified FGFR2 [11]	0.20
Ovarian	SKOV3	Over expression FGFR4 [47]	0.28
Bladder	J82	FGFR3 *5652E* [48]	0.30
Ovarian	A2780	Over expression FGFR 4 [47]	0.33
Leukemia	K-562	Over expression FGFR3 / 4 [49]	0.40
Breast	MFM-223	Amplified FGFR2 [42]	0.50
Bladder	RT112	FGFR3-TACC3 fusion [19]	0.65
Endometrial	MFE-280	FGFR2 *S252W* [50]	0.9
Endometrial	MFE-296	FGFR2 *N549K* [50]	1.0
Bladder	RT4	FGFR3-TACC3 fusion [19]	1.2
Bladder	SW-780	FGFR3-BAIAP2L1 fusion [19]	1.4
Endometrial	AN3CA	FGFR2 *N549K* [50]	1.7

ARQ 087 inhibited multiple cell lines known to have FGFR1 or FGFR3 fusions including; RT4 and RT112 (FGFR3-TACC3), SW-780 (FGFR3-BAIAP2L1), and KG-1(FGFROP2-FGFR1), with GI_50_ values between 0.13 to 1.4 μM Table 3, suggesting that cell lines containing FGFR fusions are sensitive to ARQ 087.

#### Alteration of cell cycle progression

**Fig 4:**
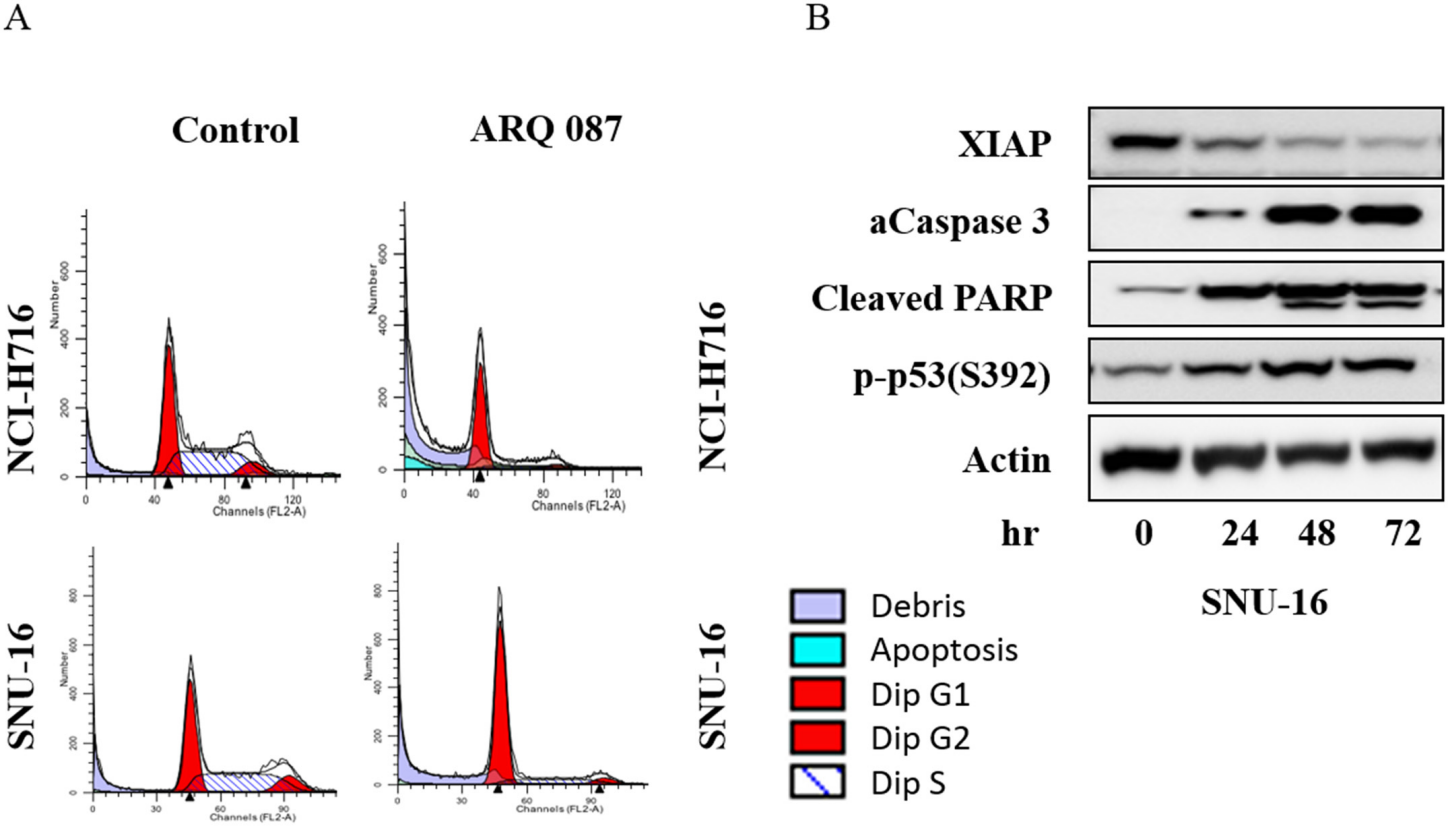
ARQ 087 arrests cells in the G1 cell cycle phase and induces apoptosis. (A) NCI-H716 and SNU-16 cells were treated with 1 μM of ARQ 087 or vehicle for 72 hours. Cell cycle profiles were measured by flow cytometric analyses. (B) SNU-16 cells were treated with ARQ 087 (1 mM) for 0, 24, 48 and 72 hrs. Western blot analysis was performed for XIAP, cleaved-PARP (c-PARP), activated-caspase 3 (a-Caspase 3), and phospho-p53 were assessed by Western blot analyses. β-Actin was used as the loading control.

The mechanism by which ARQ 087 inhibits the growth of FGFR kinase dependent cell lines was investigated. NCI-H716 and SNU-16 cells were treated with either 0.1 μM or 1 μM of ARQ 087 for 24 or 72 hours then subjected to flow cytometric analysis. Camptothecin, a known inhibitor of cell cycle progression was used as the positive control in these experiments. Treatment with ARQ 087 led to an accumulation of cells in the G1 phase cell cycle in a dose- and time-dependent manner Fig 4A and Table 4. We also found that the sub-G1 population Fig 4A of NCI-H716 cells, increased after treatment with 1 μM of ARQ 087 Fig 4A and Table 4. To investigate whether the disruption of the cell cycle correlated with an increase in apoptosis SNU-16 cells were treated with 1 μM of ARQ 087 for 0, 24, 48, and 72 hours, and X-linked inhibitor of apoptosis protein (XIAP), cleaved PARP, activated caspase 3, phospho-p53 were assessed by Western blotting analysis. We observed a decrease in XIAP, and an increase in cleaved-PARP, activated-caspase 3, and pP53 Fig 4B. Taken together, these data suggest that ARQ 087 inhibits the proliferation of FGFR amplified cells by inducing G1 cell cycle arrest and apoptosis.

**Table 4:** ARQ 087 induces a G1 cell cycle arrest in cancer cells.

**24 Hours**		**Cell Cycle (% cells)**			
Cell Line	Treatment	Sub-G1	G1	S	G2/M
NCI-H716	DMSO	1.7	35.1	42.0	21.2
	ARQ 087 0.1 μM	0.9	74.6	16.4	8.1
	ARQ 087 1.0 μM	3.1	66.5	22.7	7.8
SNU-16	DMSO	0.0	29.2	54.7	16.1
	ARQ 087 0.1 μM	0.1	37.4	49.2	13.3
	ARQ 087 1.0 μM	0.0	68.5	25.1	6.4
**72 Hours**		**Cell Cycle (% cells)**			
Cell Line	Treatment	Sub-G1	G1	S	G2/M
NCI-H716	DMSO	2.0	47.2	41.7	9.1
	ARQ 087 0.1 μM	3.8	60.9	28.0	7.3
	ARQ 087 1.0 μM	17.5	60.6	19.4	2.5
SNU-16	DMSO	0.0	41.5	46.2	12.3
	ARQ 087 0.1 μM	0.0	38.5	56.1	5.4
	ARQ 087 1.0 μM	0.0	76.7	17.5	5.7

#### *In vivo* FGFR pathway inhibition

**Fig 5:**
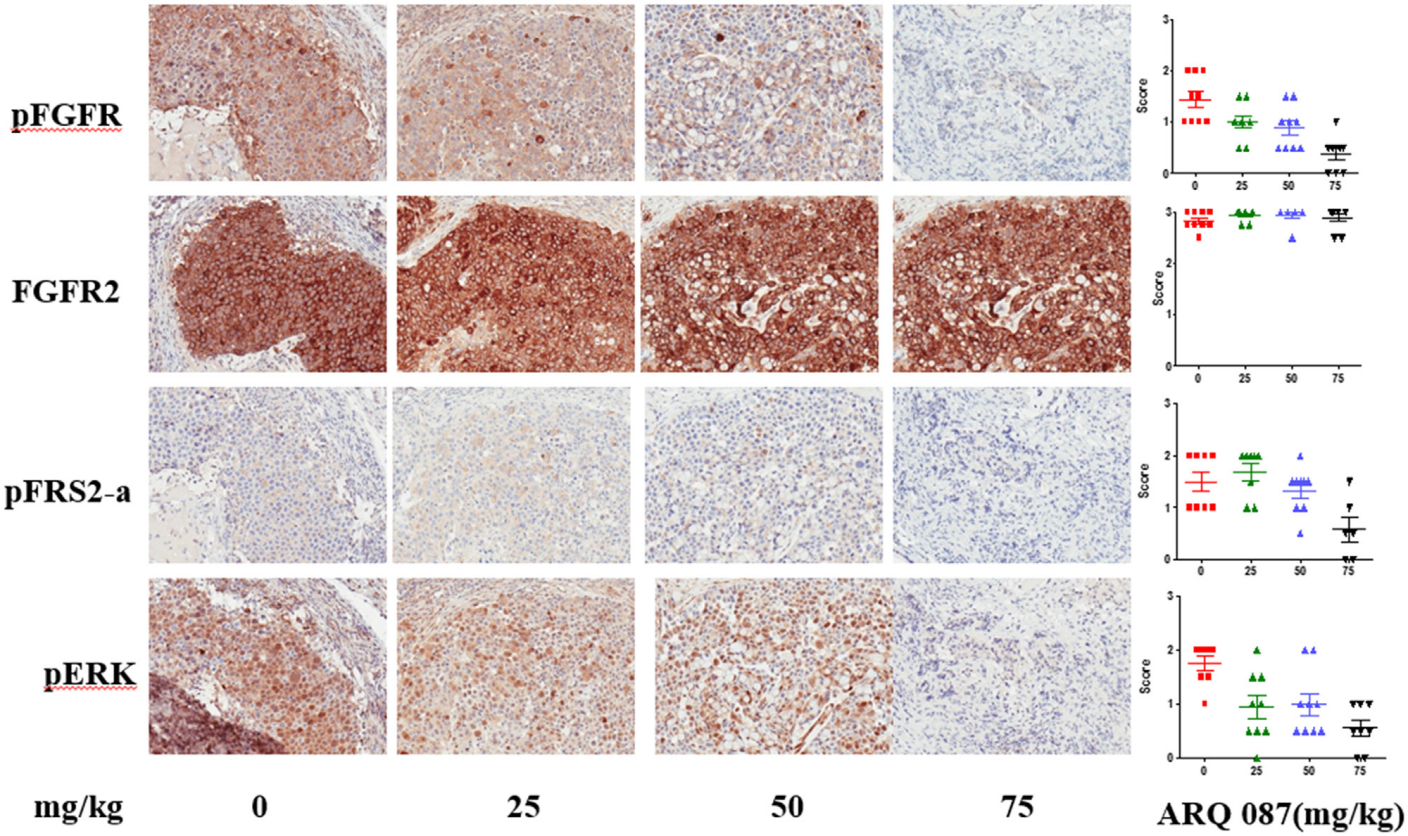
ARQ 087 inhibits the FGFR pathway in xenograft tumors. Mice were dosed with 75 mg/kg of ARQ 087, and sacrificed at day 9 after administration Tumor samples were collected 4 hr after last dose. IHC staining of pFGFR, FGFR2, pFRS2-a and pERK were performed from tumor tissues. The intensity of staining was scored by a veterinary pathologist and presented as mean±SEM. Representative photomicrographs are shown.

The *in vivo* pharmacodynamic effect of ARQ 087 was examined in SNU-16 tumor-bearing animals using IHC. Doses of 0, 25, 50, and 75 mg/kg of ARQ 087 (n = 9/group) led to a reduction in phospho-FGFR, phospho-FRS2-α, and phospho-ERK after 4 hours, while the total FGFR2 protein was unaffected by ARQ 087 treatment Fig 5. These results indicate that ARQ 087 attenuated FGFR signaling in SNU-16 human xenograft tumors.

#### *In vivo* efficacy in mouse xenograft models

**Fig 6:**
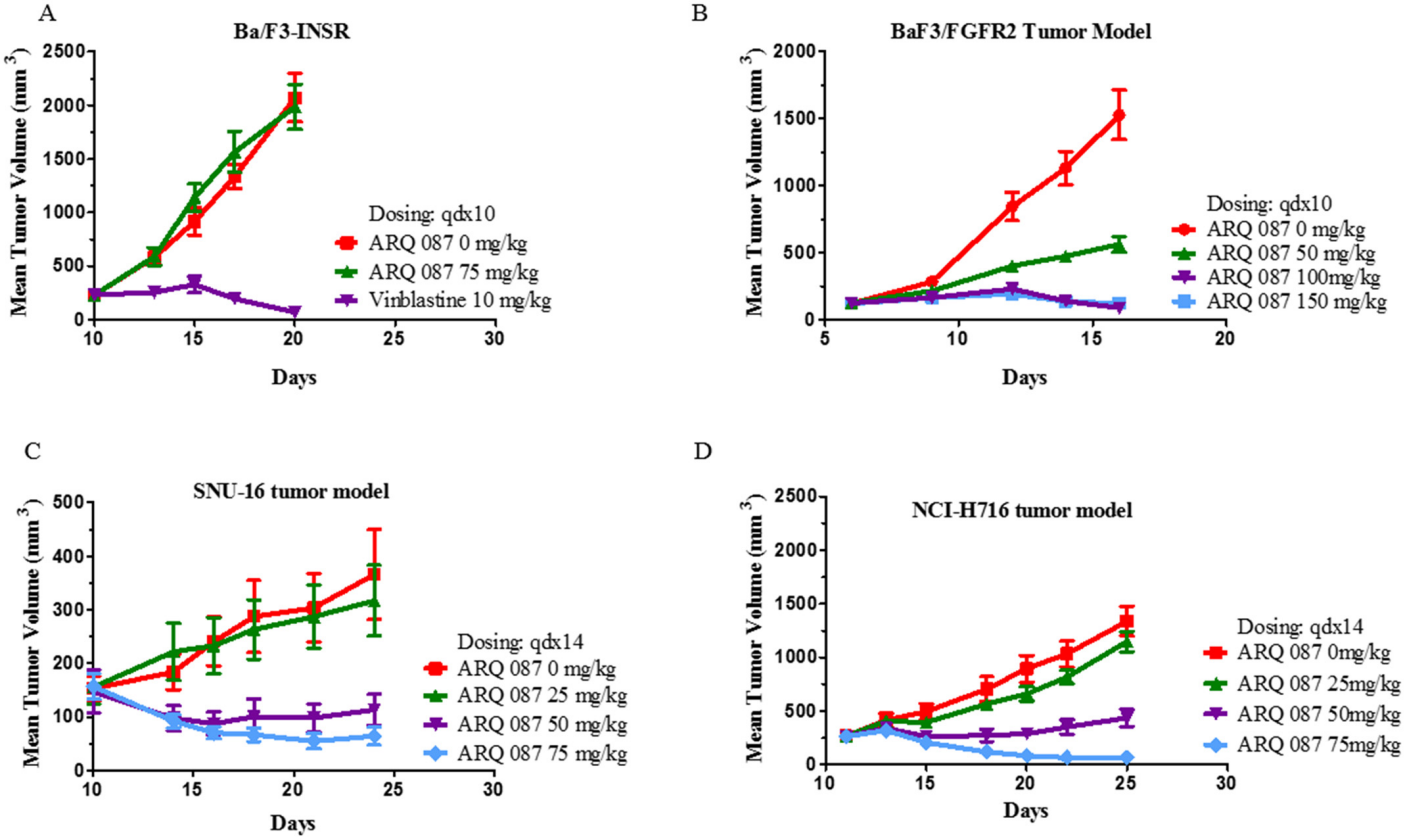
ARQ 087 activity in tumor growth models. (A, C, D), Growth is inhibited in BaF3/FGFR2, SNU-16, and NCI-H716, xenograft models, but not inhibited in (B) BaF3/INSR. Results are represented as the mean of tumor volume in mm^3^ ± SEM of each group (n = 8–10) in function of the treatment period. The tumor growth inhibition (TGI) is indicated on the plots.

**Fig 7:**
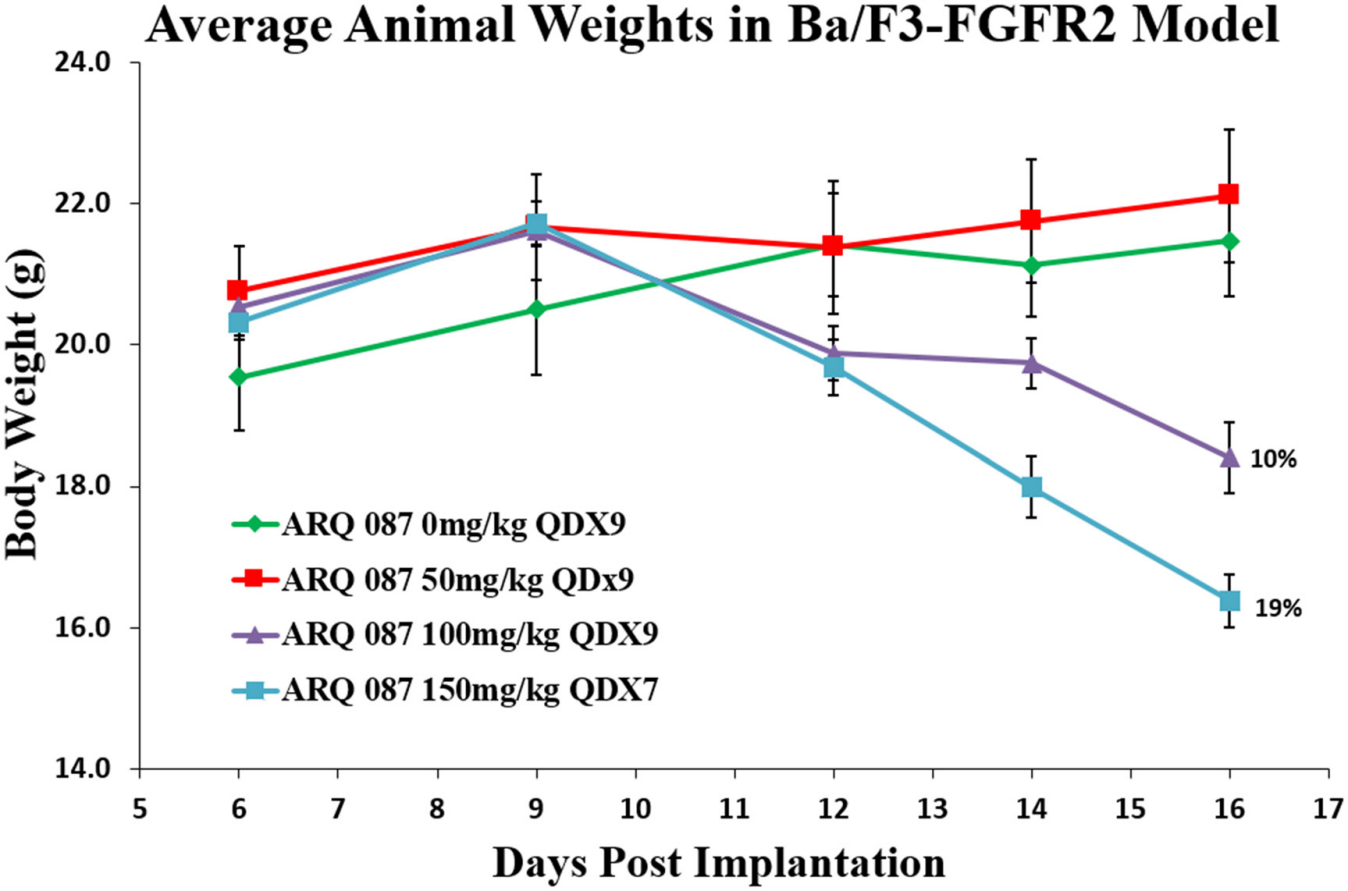
Animal weights in BaF3/FGFR2 animals dosed with ARQ 087. Mice were dosed with 0, 50, 100, and 150 mg/kg of ARQ 087, and sacrificed at 10 days after administration after the start of dosing. Mean weights +/- SEM.

The *in vivo* anti-tumor effect of ARQ 087 was assessed in athymic mice bearing Ba/F3-FGFR2, Ba/F3-INSR, SNU-16, and NCI-H716 cell line-derived tumors. The Ba/F3-FGFR2 and Ba/F3-INSR are transfected models. The SNU-16 cell line harbors amplified FGFR2 and contains a PDHX-FGFR2 fusion, while the NCI-H716, also amplified for FGFR2, contains a FGFR2-COL14A1 fusion. ARQ 087 demonstrated potent tumor growth inhibition in the Ba/F3-FGFR2 model, while failing to inhibit the growth of the Ba/F3-INSR model Fig 6A and 6B. Meaningful (>50 TGI vs. control) tumor inhibition was observed both cancer cell line derived xenograft models. In the SNU-16 xenograft study, treatment with 75 mg/kg and 50 mg/kg achieved 83% (p = 0.002) and 69% (p = 0.013) TGI, respectively Fig 6C. Partial (PR) and complete (CR) regressions also were observed in both dose groups. In the NCI-H716 human cecum model, 50 mg/kg and 75 mg/kg on a Q1Dx14 schedule demonstrated significant TGI of 68% (p = 0.0001) and 96% (p = 0.0001), respectively Fig 6D. Doses of 150 mg/kg of ARQ 087 were not well tolerated, resulting in unacceptable weight loss and general lethargy. ARQ 087 was tolerated at 100 mg/kg QD, however weight loss (~10%) prompted the adoption of 75 mg/kg for later studies Fig 7.

## Discussion

Data presented here clearly demonstrate the preclinical and clinical activity of ARQ 087, a potent pan-FGFR inhibitor showing multi-kinase activity. ARQ 087 inhibits FGFR kinase by an ATP competitive mechanism, and is capable of inhibiting both the inactive and fully active forms of the FGFR kinase. Hence, ARQ 087 delays FGFR activation by inhibiting its autophosphorylation, as well as inhibition of the phosphorylated active kinase. These data prompted further investigation of ARQ 087 using *in vitro* cell models with a genetically dysregulated FGFR2 pathway.

In cellular models, ARQ 087 was shown to inhibit FGFR phosphorylation in ectopically-expressing COS-1 cells with essentially equivalent potency for FGFR1 and FGFR2. Additionally, ARQ 087 inhibited FGFR phosphorylation in FGFR2 dependent cells. FGFR2 pathway inhibition by ARQ 087 was confirmed in multiple FGFR2 dependent cell lines further supporting FGFR2 as a target of ARQ 087.

The functional consequence of FGFR inhibition was assessed in multiple cell systems. ARQ 087 inhibited cell proliferation in Ba/F3 transfected cells which were engineered to be dependent on FGFR1-4 signaling, or on an FGFR gene fusion.

Among the cell lines that harbored FGFR fusions, amplifications, or mutations, cell lines with FGFR-fusions were among the most sensitive to ARQ 087. Likewise, Ba/F3 cell lines addicted to FGFR-fusions profiled better than the wild-type or mutant FGFR dependent Ba/F3 cells. Interestingly, a similar observation has been reported by Arai et al [34] and Wu et al. [48], where the FGFR-fusion cell lines were more sensitive to FGFR inhibitor than the amplified wild-type FGFR or mutant FGFR harboring cell lines. Furthermore, a better potency of FGFR inhibitor in cells with FGFR-fusion than wild-type or mutant is attributed to strong inhibition of ERK1/2 activation in FGFR-fusion carrying cells. We observe a similar phenomenon with FGFR-fusion harboring cells, ERK activation more potently suppressed by ARQ 087 in both NCI-H716 and SNU-16 cells than FGFR-wild type carrying KATO-III cells Fig 3B. Collectively, tumor cell lines addicted to oncogenic FGFR-fusions appear to be highly sensitive to ARQ 087.

Further investigation of the mechanism of growth inhibition in FGFR-driven tumor cell lines demonstrated that ARQ 087 induced an accumulation of cells in G1 phase of the cell cycle. Further studies suggested that ARQ 087 works through an apoptotic mechanism of cell death in the SNU-16 cell line which is FGFR2 amplified as well as harboring FGFR2 fusions.

*In vivo* assessment of pharmacodynamic biomarkers four hours after a single dose of ARQ 087 in animals with FGFR2 driven tumors showed a reduction in the phosphorylation of both FGFR and its substrate FRS2-α. Additionally the downstream marker phospho-ERK, was also shown to decrease following ARQ 087 treatment. In FGFR2-amplified tumor models where highly activated MAPK signaling (phospho-ERK staining) is driven by FGFR2 signaling, the downstream markers are also strongly inhibited by ARQ 087. These data agree well with the *in vitro* data demonstrating inhibition of the FGFR signaling pathway in these cell lines and induction of an apoptotic response.

ARQ 087 demonstrated efficacy in multiple *in vivo* xenograft models, and was well tolerated at doses up to 75 mg/kg. Durable partial and complete regressions were observed in both tumor models that are driven by FGFR2 amplification and fusions (NCI-H716 and SNU-16), and the murine transfected cell line (BaF3/FGFR2). Notably, ARQ 087 was not efficacious in a xenograft model without dependency on FGFR2: i.e. BaF3/INSR (BaF3 cells expressing the insulin receptor).

There are a number of FGF/FGFR targeted therapies currently in preclinical or clinical development, including small molecule non-selective FGFR multi-kinase inhibitors, selective FGFR inhibitors, FGFR antibodies, and FGF ligand traps [13–17]. Although there is strong preclinical data to support the targeting of FGFR [43, 51], to date preclinical success has not translated into definitive clinical success. Initial trials with small molecule, both FGFR specific and multikinase, inhibitors have shown some promising signs of antitumor response (PR/SD). However, toxicities such as hyperphosphatemia, an on-target toxicity associated with FGFR inhibition, as well as off-target toxicities such as hypertension and renal toxicities, commonly associated with VEGFR inhibitors have been observed [52–55].

The recent identification of the driver role of FGFR2 fusions in iCCA, as well as other tumor types, provides a promising path forward in the selection of patients that may respond to FGFR inhibitors in a clinical setting [20, 56]. The early experiences with FGFR inhibitors in the clinic highlight the importance of defining the appropriate patient population, and including suitable biomarker assays during clinical screening and enrollment. However, this task should become easier as the use of next generation sequencing (NGS) and FISH becomes more routine.

In conclusion, ARQ 087 is a novel and potent FGFR inhibitor with multi-kinase activity. It has the potential to be effective in variety of cancers driven by dysregulation of the FGFR pathway. Preclinical results suggest that genetic alterations of the FGFR pathway—amplification, mutation, and gene fusion, correlate with the activity of ARQ 087 *in vitro* and *in vivo*. ARQ 087 has recently completed the dose escalation part of its first in human clinical trial [NCT01752920]. A clinical response was noted for a patient with intrahepatic cholangiocarcinoma with a known FGFR2 fusion, additionally prolonged stable disease was observed in 10 of 61 patients, including a cholangiocarcinoma, which also had an FGFR2 fusion [57]. Full results from this trial will be presented at a future date. Further clinical validation is ongoing with a phase two study in FGFR2 fusion positive intrahepatic cholangiocarcinoma.

## Supporting Information

S1 Appendix animal weights and tumor volumes for *in vivo* experiments. (PDF)

S1 Table FGFR2 fusions identified in intrahepatic cholangiocarcinoma. (DOCX)
