# Supplementary material for: Preclinical Activity of ARQ 087, a Novel Inhibitor Targeting FGFR Dysregulation
Source: PLoS One. 2016 Sep 14;11(9):e0162594. doi: 10.1371/journal.pone.0162594 (PMC5023172; doi:10.1371/journal.pone.0162594)
Supplement: S1 Appendix — (PDF) [file pone.0162594.s001.pdf]

tumor:

Tumor: NCI-H716

Group #: 2 Compound: ARQ 087 Dosage: 0 mg/kg Route: PO Schedule: Q1Dx14 (SD)

Gross Animal Body Weights (g)

Gross Animal Body Weights on Days Indicated

Fate	Day 11	Day 13	Day 15	Day 18	Day 20	Day 22	Day 25
**1**	19.00	19.00	19.00	19.00	19.00	20.00	20.00
**2**	18.00	17.00	17.00	16.00	15.00	16.00	17.00
**3**	19.00	18.00	18.00	19.00	19.00	19.00	19.00
**4**	19.00	18.00	19.00	19.00	19.00	19.00	20.00
**5**	18.00	18.00	18.00	18.00	19.00	19.00	18.00
**6**	19.00	19.00	19.00	19.00	19.00	20.00	20.00
**7**	21.00	20.00	21.00	21.00	21.00	21.00	21.00
**8**	22.00	22.00	22.00	22.00	22.00	23.00	23.00
**9**	20.00	20.00	20.00	19.00	19.00	19.00	19.00
**10**	20.00	20.00	20.00	20.00	20.00	20.00	20.00
**MEAN**	19.50	19.10	19.30	19.20	19.20	19.60	19.70

Group #: 3 Compound: ARQ 087 Dosage: 25 mg/kg Route: PO Schedule: Q1Dx14 (SD)

Gross Animal Body Weights (g)

Gross Animal Body Weights on Days Indicated

Fate	Day 11	Day 13	Day 15	Day 18	Day 20	Day 22	Day 25
**1**	18.00	19.00	19.00	19.00	19.00	20.00	19.00
**2**	20.00	20.00	20.00	20.00	21.00	21.00	21.00
**3**	19.00	19.00	19.00	19.00	19.00	20.00	19.00
**4**	16.00	16.00	16.00	16.00	16.00	17.00	17.00
**5**	17.00	17.00	17.00	17.00	18.00	18.00	19.00
**6**	21.00	21.00	21.00	22.00	22.00	23.00	22.00
**7**	20.00	19.00	20.00	20.00	19.00	20.00	20.00
**8**	20.00	20.00	20.00	20.00	20.00	20.00	21.00
**9**	23.00	22.00	22.00	23.00	22.00	22.00	23.00
**10**	22.00	22.00	21.00	21.00	21.00	21.00	21.00
**MEAN**	19.60	19.50	19.50	19.70	19.70	20.20	20.20

Group #: 4 Compound: ARQ 087 Dosage: 50 mg/kg Route: PO Schedule: Q1Dx14 (SD)

Gross Animal Body Weights (g)

Gross Animal Body Weights on Days Indicated

Fate	Day 11	Day 13	Day 15	Day 18	Day 20	Day 22	Day 25
**1**	17.00	17.00	17.00	17.00	17.00	17.00	18.00
**2**	19.00	19.00	18.00	19.00	18.00	18.00	19.00
**3**	18.00	18.00	19.00	19.00	19.00	19.00	19.00
**4**	19.00	19.00	19.00	20.00	20.00	20.00	20.00
**5**	20.00	20.00	20.00	20.00	20.00	20.00	20.00
**6**	18.00	18.00	19.00	18.00	18.00	19.00	18.00
**7**	19.00	18.00	19.00	18.00	19.00	19.00	19.00
**8**	19.00	19.00	20.00	20.00	20.00	20.00	21.00
**9**	18.00	18.00	18.00	18.00	18.00	19.00	18.00
**10**	19.00	19.00	20.00	18.00	19.00	19.00	20.00
**MEAN**	18.60	18.50	18.90	18.70	18.80	19.00	19.20

Group #: 5 Compound: ARQ 087 Dosage: 75 mg/kg Route: PO Schedule: Q1Dx14 (SD)

Gross Animal Body Weights (g)

Gross Animal Body Weights on Days Indicated

Fate	Day 11	Day 13	Day 15	Day 18	Day 20	Day 22	Day 25
**1**	19.00	19.00	19.00	19.00	19.00	19.00	20.00
**2**	18.00	17.00	17.00	17.00	17.00	17.00	17.00
**3**	18.00	18.00	18.00	18.00	18.00	18.00	18.00
**4**	20.00	20.00	20.00	20.00	20.00	20.00	20.00
**5**	18.00	18.00	18.00	18.00	17.00	17.00	17.00
**6**	19.00	19.00	18.00	19.00	19.00	19.00	19.00

**7**	19.00	19.00	18.00	18.00	18.00	18.00	17.00
**8**	18.00	17.00	17.00	17.00	17.00	18.00	17.00
**9**	20.00	20.00	20.00	19.00	20.00	20.00	20.00
**10**	18.00	18.00	18.00	18.00	18.00	18.00	18.00
**MEAN**	18.70	18.50	18.30	18.30	18.30	18.40	18.30

Group Details (Tumors)

Tumor: NCI-H716

Group #: 2 Compound: ARQ 087 Dosage: 0 mg/kg Route: PO Schedule: Q1Dx14 (SD)

Tumor Weights (mg): Tumor Volume In Milligrams On Days Indicated

Fate	Day 11	Day 13	Day 15	Day 18	Day 20	Day 22	Day 25
**1**	245.00	289.00	389.00	545.00	715.00	996.00	1384.00
**2**	150.00	172.00	169.00	227.00	423.00	606.00	815.00
**3**	334.00	520.00	568.00	685.00	1133.00	1221.00	1524.00
**4**	452.00	741.00	849.00	1064.00	1313.00	1447.00	1721.00
**5**	174.00	282.00	295.00	519.00	654.00	778.00	1020.00
**6**	185.00	284.00	488.00	676.00	739.00	887.00	1525.00
**7**	259.00	365.00	460.00	581.00	759.00	947.00	1261.00
**8**	397.00	759.00	862.00	1433.00	1507.00	1739.00	1955.00
**9**	318.00	440.00	571.00	973.00	1317.00	1214.00	1655.00
**10**	138.00	286.00	292.00	341.00	372.00	487.00	503.00
**MEAN**	265.20	413.80	494.30	704.40	893.20	1032.20	1336.30
**MEDIAN**	252.00	327.00	474.00	628.50	749.00	971.50	1454.00
**STANDARD ERROR**	34.06	63.70	72.35	113.94	125.33	120.81	140.53

Group #: 3 Compound: ARQ 087 Dosage: 25 mg/kg Route: PO Schedule: Q1Dx14 (SD)

Tumor Weights (mg): Tumor Volume In Milligrams On Days Indicated

Fate	Day 11	Day 13	Day 15	Day 18	Day 20	Day 22	Day 25
**1**	216.00	283.00	329.00	553.00	631.00	868.00	1183.00
**2**	280.00	328.00	413.00	499.00	596.00	710.00	1004.00
**3**	356.00	523.00	595.00	653.00	696.00	1133.00	1159.00
**4**	355.00	528.00	535.00	583.00	706.00	817.00	1201.00
**5**	216.00	356.00	335.00	427.00	562.00	719.00	1421.00
**6**	227.00	515.00	418.00	896.00	1073.00	1103.00	1586.00
**7**	223.00	370.00	360.00	470.00	499.00	768.00	1057.00
**8**	153.00	289.00	235.00	395.00	449.00	526.00	723.00
**9**	434.00	555.00	444.00	719.00	933.00	974.00	1469.00
**10**	175.00	298.00	286.00	456.00	446.00	553.00	677.00
**MEAN**	263.50	404.50	395.00	565.10	659.10	817.10	1148.00
**MEDIAN**	225.00	363.00	386.50	526.00	613.50	792.50	1171.00
**STANDARD ERROR**	28.64	35.43	34.88	48.84	64.92	65.55	94.58

Group #: 4 Compound: ARQ 087 Dosage: 50 mg/kg Route: PO Schedule: Q1Dx14 (SD)

Tumor Weights (mg): Tumor Volume In Milligrams On Days Indicated

Fate	Day 11	Day 13	Day 15	Day 18	Day 20	Day 22	Day 25
**1**	189.00	201.00	127.00	135.00	173.00	186.00	236.00
**2**	247.00	329.00	261.00	231.00	317.00	301.00	414.00
**3**	252.00	219.00	165.00	179.00	240.00	214.00	344.00
**4**	397.00	694.00	646.00	785.00	708.00	893.00	1105.00
**5**	279.00	340.00	295.00	270.00	337.00	424.00	377.00
**6**	156.00	240.00	152.00	139.00	159.00	194.00	180.00
**7**	351.00	343.00	239.00	236.00	254.00	342.00	416.00
**8**	295.00	336.00	233.00	296.00	317.00	383.00	551.00
**9**	233.00	398.00	232.00	207.00	162.00	322.00	355.00
**10**	215.00	296.00	277.00	251.00	224.00	248.00	361.00
**MEAN**	261.40	339.60	262.70	272.90	289.10	350.70	433.90
**MEDIAN**	249.50	332.50	236.00	233.50	247.00	311.50	369.00
**STANDARD ERROR**	22.96	44.06	46.04	59.30	50.97	65.39	81.06

Group #: 5 Compound: ARQ 087 Dosage: 75 mg/kg Route: PO Schedule: Q1Dx14 (SD)

Tumor Weights (mg): Tumor Volume In Milligrams On Days Indicated

Fate	Day 11	Day 13	Day 15	Day 18	Day 20	Day 22	Day 25
**1**	309.00	334.00	154.00	83.00	69.00	49.00	45.00
**2**	378.00	483.00	340.00	277.00	127.00	118.00	66.00
**3**	152.00	271.00	138.00	58.00	72.00	57.00	56.00
**4**	169.00	208.00	144.00	84.00	80.00	63.00	88.00
**5**	314.00	406.00	222.00	124.00	89.00	69.00	70.00
**6**	196.00	251.00	185.00	92.00	81.00	57.00	94.00
**7**	195.00	278.00	141.00	58.00	49.00	60.00	57.00
**8**	408.00	414.00	387.00	206.00	128.00	80.00	56.00
**9**	194.00	195.00	103.00	102.00	89.00	62.00	97.00
**10**	307.00	362.00	226.00	88.00	54.00	34.00	40.00
**MEAN**	262.20	320.20	204.00	117.20	83.80	64.90	66.90
**MEDIAN**	251.50	306.00	169.50	90.00	80.50	61.00	61.50
**STANDARD ERROR**	29.05	30.16	29.40	22.23	8.40	7.02	6.36

Group Details (Gross Weights)

Tumor: SNU-16

Group #: 2 Compound: ARQ 087 Dosage: 0 mg/kg Route: PO Schedule: Q1Dx14 (SD)

Gross Animal Body Weights (g)

Gross Animal Body Weights on Days Indicated						
	**Day 10**	**Day 14**	**Day 16**	**Day 18**	**Day 21**	**Day 24**
**1**	25.00	26.00	27.00	27.00	28.00	27.00
**2**	22.00	22.00	23.00	23.00	24.00	24.00
**3**	22.00	23.00	24.00	24.00	25.00	25.00
**4**	25.00	24.00	25.00	25.00	26.00	26.00
**5**	21.00	21.00	23.00	23.00	23.00	23.00
**6**	21.00	21.00	22.00	24.00	24.00	25.00
**7**	21.00	21.00	22.00	22.00	23.00	23.00
**8**	22.00	22.00	23.00	23.00	25.00	25.00
**MEAN**	22.38	22.50	23.63	23.88	24.75	24.75

Group #: 3 Compound: ARQ 087 Dosage: 75 mg/kg Route: PO Schedule: Q1Dx14 (SD)

Gross Animal Body Weights (g)

Gross Animal Body Weights on Days Indicated						
	**Day 10**	**Day 14**	**Day 16**	**Day 18**	**Day 21**	**Day 24**
**1**	22.00	22.00	23.00	24.00	25.00	25.00
**2**	21.00	21.00	22.00	22.00	22.00	22.00
**3**	24.00	24.00	25.00	26.00	24.00	25.00
**4**	18.00	18.00	18.00	18.00	19.00	18.00
**5**	23.00	23.00	23.00	23.00	24.00	24.00
**6**	22.00	23.00	23.00	22.00	23.00	23.00
**7**	22.00	23.00	23.00	24.00	25.00	23.00
**8**	23.00	22.00	22.00	22.00	23.00	21.00
**9**	22.00	22.00	22.00	22.00	24.00	23.00
**MEAN**	21.89	22.00	22.33	22.56	23.22	22.67

Group #: 4 Compound: ARQ 087 Dosage: 50 mg/kg Route: PO Schedule: Q1Dx14 (SD)

Gross Animal Body Weights (g)

Gross Animal Body Weights on Days Indicated						
	**Day 10**	**Day 14**	**Day 16**	**Day 18**	**Day 21**	**Day 24**
**1**	20.00	21.00	20.00	21.00	22.00	23.00
**2**	22.00	22.00	23.00	23.00	24.00	23.00
**3**	20.00	21.00	21.00	22.00	23.00	23.00
**4**	21.00	21.00	22.00	22.00	22.00	23.00
**5**	21.00	22.00	23.00	23.00	23.00	23.00
**6**	25.00	25.00	26.00	26.00	25.00	23.00
**7**	23.00	24.00	23.00	24.00	23.00	24.00
**8**	21.00	21.00	21.00	22.00	22.00	22.00
**MEAN**	21.63	22.13	22.38	22.88	23.00	23.00

Group #: 5 Compound: ARQ 087 Dosage: 25 mg/kg Route: PO Schedule: Q1Dx14 (SD)

Gross Animal Body Weights (g)

Gross Animal Body Weights on Days Indicated						
	**Day 10**	**Day 14**	**Day 16**	**Day 18**	**Day 21**	**Day 24**
**1**	20.00	21.00	22.00	22.00	22.00	22.00
**2**	21.00	22.00	23.00	23.00	23.00	24.00
**3**	23.00	24.00	24.00	24.00	25.00	24.00
**4**	23.00	24.00	26.00	26.00	26.00	27.00
**5**	21.00	22.00	23.00	24.00	24.00	24.00
**6**	22.00	22.00	23.00	23.00	24.00	24.00
**7**	22.00	23.00	23.00	23.00	24.00	23.00
**8**	22.00	22.00	24.00	23.00	24.00	23.00
**MEAN**	21.75	22.50	23.50	23.50	24.00	23.88

Group Details (Tumors)

Tumor: SNU-16

Group #: 2 Compound: ARQ 087 Dosage: 0 mg/kg Route: PO Schedule: Q1Dx14 (SD)

Tumor Weights (mg):

	Tumor Volume In .	.	.	.	.	
	**Day 10**	**Day 14**	**Day 16**	**Day 18**	**Day 21**	**Day 24**
**1**	185.00	288.00	335.00	329.00	408.00	461.00
**2**	92.00	109.00	127.00	116.00	112.00	89.00
**3**	141.00	189.00	171.00	207.00	307.00	312.00
**4**	66.00	62.00	64.00	67.00	50.00	56.00
**5**	368.00	559.00	543.00	584.00	586.00	618.00
**6**	134.00	220.00	249.00	296.00	325.00	412.00
**7**	120.00	169.00	159.00	228.00	248.00	308.00
**8**	139.00	176.00	212.00	277.00	260.00	279.00
**MEAN**	155.63	221.50	232.50	263.00	287.00	316.88
**MEDIAN**	136.50	182.50	191.50	252.50	283.50	310.00
**STANDARD ERROR**	32.81	53.84	52.83	55.59	59.00	65.74

Group #: 3 Compound: ARQ 087 Dosage: 75 mg/kg Route: PO Schedule: Q1Dx14 (SD)

Tumor Weights (mg):

	Tumor Volume In .	.	.	.	.		
	**Day 10**	**Day 14**	**Day 16**	**Day 18**	**Day 21**	**Day 24**	
**1**	111.00	98.00	92.00	96.00	41.00	52.00	
**2**	171.00	104.00	68.00	77.00	80.00	115.00	
**3**	289.00	104.00	42.00	14.00	0.00	0.00	
**4**	167.00	137.00	137.00	135.00	111.00	150.00	
**5**	81.00	34.00	37.00	28.00	14.00	37.00	79.80276134
**6**	67.00	39.00	39.00	28.00	14.00	14.00	83.22580645
**7**	205.00	94.00	44.00	65.00	117.00	113.00	
**8**	99.00	82.00	77.00	80.00	59.00	41.00	
**9**	219.00	135.00	94.00	77.00	53.00	54.00	
**MEAN**	156.56	91.89	70.00	66.67	54.33	64.00	
**MEDIAN**	167.00	98.00	68.00	77.00	53.00	52.00	
**STANDARD ERROR**	24.50	12.06	11.25	12.74	14.05	16.87	

Group #: 4 Compound: ARQ 087 Dosage: 50 mg/kg Route: PO Schedule: Q1Dx14 (SD)

Tumor Weights (mg):

	Tumor Volume In .	.	.	.	.	
	**Day 10**	**Day 14**	**Day 16**	**Day 18**	**Day 21**	**Day 24**
**1**	131.00	79.00	74.00	53.00	52.00	64.00
**2**	152.00	141.00	116.00	146.00	157.00	155.00
**3**	77.00	44.00	40.00	27.00	30.00	38.00
**4**	113.00	95.00	81.00	78.00	90.00	128.00
**5**	75.00	42.00	42.00	52.00	66.00	89.00
**6**	73.00	31.00	27.00	14.00	14.00	14.00
**7**	418.00	231.00	216.00	303.00	227.00	275.00
**8**	136.00	116.00	105.00	127.00	149.00	144.00
**MEAN**	146.88	97.38	87.63	100.00	98.13	113.38
**MEDIAN**	122.00	87.00	77.50	65.50	78.00	108.50
**STANDARD ERROR**	40.21	23.44	21.48	33.21	25.91	29.15

Group #: 5 Compound: ARQ 087 Dosage: 25 mg/kg Route: PO Schedule: Q1Dx14 (SD)

Tumor Weights (mg):

	Tumor Volume In .	.	.	.	.	
	**Day 10**	**Day 14**	**Day 16**	**Day 18**	**Day 21**	**Day 24**
**1**	62.00	52.00	36.00	27.00	38.00	55.00
**2**	128.00	215.00	368.00	477.00	495.00	444.00
**3**	108.00	299.00	365.00	329.00	451.00	530.00
**4**	139.00	144.00	179.00	205.00	229.00	265.00
**5**	243.00	178.00	271.00	295.00	333.00	403.00
**6**	264.00	325.00	394.00	620.00	532.00	801.00
**7**	154.00	111.00	142.00	125.00	113.00	121.00
**8**	123.00	147.00	172.00	219.00	237.00	309.00
**MEAN**	152.63	183.88	240.88	287.13	303.50	366.00
**MEDIAN**	133.50	162.50	225.00	257.00	285.00	356.00
**STANDARD ERROR**	24.07	32.68	45.56	67.35	63.84	83.85

Group Details (Gross Weights)

Tumor: Baf3/FGFR2

Group #: 1 Compound: ARQ 087 Dosage: 0 mg/kg Route: PO Schedule: Q1DX9 (SD+1)

Gross Animal Body Weights (g)

	Gross Animal Body Weights on Days Indicated					
	**Fate**	**Day 6**	**Day 9**	**Day 12**	**Day 14**	**Day 16**
**1**		20.00	22.00	24.00	24.00	25.00
**2**		21.00	22.00	23.00	22.00	23.00
**3**		24.00	25.00	26.00	26.00	27.00
**4**		21.00	22.00	22.00	22.00	23.00
**5**		19.00	20.00	21.00	21.00	22.00
**6**		18.00	19.00	20.00	20.00	20.00
**7**		18.00	20.00	20.00	20.00	21.00
**8**		20.00	22.00	22.00	23.00	23.00
**9**	ACC12	16.00	15.00			
**MEAN**		19.67	20.78	22.25	22.25	23.00

Group #: 3 Compound: ARQ 087 Dosage: 150 mg/kg Route: PO Schedule: Q1DX9 (SD+1)

Gross Animal Body Weights (g)

	Gross Animal Body Weights on Days Indicated					
	**Fate**	**Day 6**	**Day 9**	**Day 12**	**Day 14**	**Day 16**
**1**		21.00	23.00	21.00	20.00	18.00
**2**		20.00	22.00	20.00	18.00	16.00
**3**		20.00	21.00	19.00	18.00	17.00
**4**		22.00	23.00	21.00	19.00	17.00
**5**	ACC12	20.00	22.00			
**6**		20.00	21.00	20.00	18.00	17.00
**7**		20.00	21.00	19.00	17.00	15.00
**8**		20.00	21.00	18.00	16.00	15.00
**9**		21.00	23.00	21.00	19.00	17.00
**MEAN**		20.44	21.89	19.88	18.13	16.50

Group #: 4 Compound: ARQ 087 Dosage: 100 mg/kg Route: PO Schedule: Q1DX9 (SD+1)

Gross Animal Body Weights (g)

	Gross Animal Body Weights on Days Indicated					
	**Fate**	**Day 6**	**Day 9**	**Day 12**	**Day 14**	**Day 16**
**1**		21.00	22.00	20.00	20.00	17.00
**2**	DEA14	19.00	21.00	18.00		
**3**		21.00	21.00	20.00	20.00	19.00
**4**		20.00	22.00	20.00	21.00	21.00
**5**		20.00	21.00	19.00	19.00	17.00
**6**		21.00	22.00	21.00	20.00	19.00
**7**		21.00	22.00	20.00	18.00	17.00
**8**		21.00	22.00	22.00	20.00	19.00
**9**		22.00	23.00	21.00	21.00	19.00
**MEAN**		20.67	21.78	20.11	19.88	18.50

Group #: 5 Compound: ARQ 087 Dosage: 50 mg/kg Route: PO Schedule: Q1Dx9 (SD+1)

Gross Animal Body Weights (g)

	Gross Animal Body Weights on Days Indicated					
	**Fate**	**Day 6**	**Day 9**	**Day 12**	**Day 14**	**Day 16**
**1**		25.00	27.00	28.00	28.00	29.00
**2**		20.00	21.00	21.00	21.00	22.00
**3**		21.00	22.00	22.00	23.00	23.00
**4**		22.00	23.00	23.00	24.00	24.00
**5**		19.00	20.00	18.00	19.00	19.00
**6**		20.00	21.00	21.00	21.00	22.00
**7**		22.00	22.00	22.00	22.00	22.00
**8**		20.00	22.00	22.00	22.00	23.00
**9**		19.00	19.00	19.00	20.00	20.00
**MEAN**		20.89	21.89	21.78	22.22	22.67

Group Details (Tumors)

Tumor: Baf3/FGFR2

Group #: 1 Compound: ARQ 087 Dosage: 0 mg/kg Route: PO Schedule: Q1DX9 (SD+1)

Tumor Weights (mg):

	Tumor Volume In	.	.	.	
**Fate**	**Day 6**	**Day 9**	**Day 12**	**Day 14**	**Day 16**
**1**	118.00	370.00	1323.00	1700.00	2554.00
**2**	112.00	315.00	820.00	906.00	1359.00
**3**	107.00	256.00	537.00	710.00	1010.00
**4**	106.00	242.00	538.00	879.00	868.00
**5**	73.00	253.00	703.00	1051.00	1647.00
**6**	135.00	267.00	795.00	1294.00	1769.00
**7**	169.00	233.00	1280.00	1577.00	1715.00
**8**	125.00	211.00	753.00	927.00	1298.00
**9** ACC12	156.00	396.00			
**MEAN**	122.33	282.56	843.63	1130.50	1527.50
**MEDIAN**	118.00	256.00	774.00	989.00	1503.00

Group #: 3 Compound: ARQ 087 Dosage: 150 mg/kg Route: PO Schedule: Q1DX9 (SD+1)

Tumor Weights (mg):

	Tumor Volume In	.	.	.	
**Fate**	**Day 6**	**Day 9**	**Day 12**	**Day 14**	**Day 16**
**1**	131.00	201.00	270.00	237.00	303.00
**2**	218.00	301.00	240.00	222.00	177.00
**3**	73.00	72.00	202.00	147.00	106.00
**4**	101.00	174.00	0.00	0.00	0.00
**5** ACC12	112.00	91.00			
**6**	98.00	163.00	231.00	126.00	84.00
**7**	171.00	240.00	267.00	252.00	198.00
**8**	86.00	138.00	219.00	97.00	104.00
**9**	94.00	97.00	96.00	14.00	0.00
**MEAN**	120.44	164.11	190.63	136.88	121.50
**MEDIAN**	101.00	163.00	225.00	136.50	105.00

Group #: 4 Compound: ARQ 087 Dosage: 100 mg/kg Route: PO Schedule: Q1DX9 (SD+1)

Tumor Weights (mg):

	Tumor Volume In	.	.	.	
**Fate**	**Day 6**	**Day 9**	**Day 12**	**Day 14**	**Day 16**
**1**	147.00	132.00	207.00	181.00	171.00
**2** DEA14	174.00	250.00	368.00		
**3**	97.00	157.00	260.00	186.00	140.00
**4**	112.00	252.00	295.00	208.00	200.00
**5**	90.00	0.00	0.00	0.00	0.00
**6**	106.00	171.00	285.00	145.00	110.00
**7**	155.00	192.00	154.00	14.00	0.00
**8**	100.00	148.00	256.00	256.00	0.00
**9**	139.00	172.00	246.00	129.00	72.00
**MEAN**	124.44	163.78	230.11	139.88	86.63
**MEDIAN**	112.00	171.00	256.00	163.00	91.00

Group #: 5 Compound: ARQ 087 Dosage: 50 mg/kg Route: PO Schedule: Q1Dx9 (SD+1)

Tumor Weights (mg):

	Tumor Volume In	.	.	.	
**Fate**	**Day 6**	**Day 9**	**Day 12**	**Day 14**	**Day 16**
**1**	95.00	123.00	225.00	363.00	458.00
**2**	104.00	187.00	390.00	406.00	568.00
**3**	197.00	367.00	613.00	703.00	713.00
**4**	122.00	233.00	392.00	490.00	649.00
**5**	134.00	213.00	466.00	638.00	927.00
**6**	122.00	211.00	437.00	512.00	470.00
**7**	110.00	142.00	377.00	376.00	458.00
**8**	149.00	277.00	444.00	463.00	458.00
**9**	115.00	184.00	257.00	306.00	343.00
**MEAN**	127.56	215.22	400.11	473.00	560.44
**MEDIAN**	122.00	211.00	392.00	463.00	470.00

Weight Data

Tumor: Baf3/INSR

Group #: 2 Compound: ARQ 087 Dosage: 0 mg/kg Schedule: Q1D x 10(SD)

			Gross Animal				
	**Fate**		**Day 10**	**Day 13**	**Day 15**	**Day 17**	**Day 20**
**1**			21.00	21.00	22.00	23.00	23.00
**2**			23.00	23.00	23.00	24.00	25.00
**3**			21.00	22.00	22.00	22.00	23.00
**4**			25.00	25.00	26.00	26.00	26.00
**5**			24.00	25.00	25.00	25.00	25.00
**6**			24.00	25.00	25.00	25.00	26.00
**7**			24.00	25.00	25.00	25.00	26.00
**8**			20.00	21.00	21.00	22.00	23.00
**9**			22.00	23.00	23.00	24.00	25.00
**10**			22.00	22.00	22.00	22.00	22.00

Group #: 3 Compound: ARQ 087 Dosage: 75 mg/kg Schedule: Q1D x 10(SD)

			Gross Animal				
	**Fate**		**Day 10**	**Day 13**	**Day 15**	**Day 17**	**Day 20**
**1**			22.00	23.00	23.00	23.00	23.00
**2**			23.00	23.00	24.00	23.00	23.00
**3**			22.00	23.00	23.00	23.00	25.00
**4**			22.00	22.00	22.00	22.00	22.00
**5**			25.00	25.00	26.00	26.00	25.00
**6**			19.00	20.00	20.00	20.00	21.00
**7**			21.00	22.00	22.00	23.00	24.00
**8**			26.00	28.00	28.00	28.00	28.00
**9**			23.00	24.00	24.00	24.00	26.00
**10**			21.00	22.00	22.00	22.00	23.00

Group #: 4 Compound: Vinblastine Dosage: 10 mg/kg Schedule: Q5Dx2(SD)

			Gross Animal				
	**Fate**		**Day 10**	**Day 13**	**Day 15**	**Day 17**	**Day 20**
**1**			22.00	21.00	21.00	20.00	21.00
**2**			22.00	21.00	22.00	21.00	23.00
**3**			24.00	24.00	24.00	23.00	24.00
**4**			22.00	21.00	21.00	20.00	21.00
**5**			26.00	24.00	25.00	24.00	24.00
**6**			22.00	22.00	22.00	22.00	22.00
**7**			20.00	19.00	20.00	19.00	19.00
**8**	SAC17		23.00	22.00	21.00	20.00	
**9**			23.00	23.00	23.00	22.00	23.00
**10**			22.00	20.00	21.00	20.00	21.00

Weight Data

Tumor: Baf3/INSR

Group #: 2 Compound: ARQ 087 Dosage: 0 mg/kg Route: PO Schedule: Q1D x 10 (SD)

Tumor Weights (mg):

	Fate	s to 2 Doublings		Day 10	Day 13	Day 15	Day 17	Day 20
				Tumor Volum .		.	.	.
1		5.9		338.00	618.00	1127.00	1656.00	2939.00
2		6.3		286.00	617.00	817.00	1376.00	2518.00
3		4.1		165.00	510.00	833.00	1149.00	1736.00
4		1.9		97.00	908.00	1420.00	1790.00	3117.00
5		3.4		228.00	771.00	1647.00	1865.00	2609.00
6		6.0		229.00	434.00	698.00	1199.00	1680.00
7		7.0		231.00	500.00	518.00	923.00	1150.00
8		6.0		296.00	684.00	979.00	1433.00	1927.00
9		5.9		237.00	422.00	823.00	1123.00	2027.00
10		6.8		186.00	359.00	290.00	843.00	1002.00
**MEAN**				229.30	582.30	915.20	1335.70	2070.50
**MEDIAN**				230.00	563.50	828.00	1287.50	1977.00
**STANDARD ERROR**				21.87	54.17	127.37	111.16	226.46
**T/C**				99.87	95.49	104.58	78.80	84.90

Group #: 3 Compound: ARQ 087 Dosage: 75 mg/kg Route: PO Schedule: Q1D x 10 (SD)

Tumor Weights (mg):

	Fate	s to 2 Doublings		Day 10	Day 13	Day 15	Day 17	Day 20
				Tumor Volum .		.	.	.
1		5.6		325.00	701.00	1237.00	1469.00	1688.00
2		3.9		176.00	511.00	1013.00	1407.00	1948.00
3		5.4		341.00	759.00	1230.00	2210.00	2068.00
4		8.5		141.00	151.00	358.00	406.00	785.00
5		4.6		285.00	783.00	1235.00	1884.00	2295.00
6		4.8		171.00	203.00	763.00	1036.00	1617.00
7		3.6		271.00	895.00	1676.00	2500.00	3407.00
8		2.0		108.00	830.00	1729.00	1914.00	2085.00
9		4.8		238.00	431.00	1032.00	1664.00	2298.00
10		4.4		236.00	636.00	1131.00	1160.00	1658.00
**MEAN**				229.20	590.00	1140.40	1565.00	1984.90
**MEDIAN**				237.00	668.50	1180.50	1566.50	2008.00
**STANDARD ERROR**				24.80	81.98	126.59	192.78	210.95
**T/C**				99.83	96.75	130.32	92.32	81.39

Group #: 4 Compound: Vinblastine Dosage: 10 mg/kg Route: IV Schedule: Q5Dx2 (SD)

Tumor Weights (mg):

	Fate	s to 2 Doublings		Day 10	Day 13	Day 15	Day 17	Day 20
				Tumor Volum				
1		NA		86.00	121.00	99.00	42.00	0.00
2		NA		263.00	158.00	252.00	114.00	90.00
3		NA		156.00	140.00	248.00	110.00	14.00
4		NA		358.00	361.00	252.00	118.00	14.00
5		NA		161.00	237.00	142.00	346.00	14.00
6		NA		287.00	252.00	388.00	334.00	300.00
7		NA		258.00	383.00	343.00	14.00	0.00
8	SAC17	>7.0		323.00	595.00	955.00	484.00	
9		NA		286.00	230.00	424.00	352.00	191.00
10		NA		129.00	90.00	156.00	52.00	0.00
**MEAN**				230.70	256.70	325.90	196.60	69.22
**MEDIAN**				260.50	233.50	252.00	116.00	14.00
**STANDARD ERROR**				28.76	48.53	77.51	52.35	35.75
**T/C**				100.48	42.10	37.24	11.60	2.84
