# Supplementary material for: Preclinical Activity of ARQ 087, a Novel Inhibitor Targeting FGFR Dysregulation
Source: PLoS One. 2016 Sep 14;11(9):e0162594. doi: 10.1371/journal.pone.0162594 (PMC5023172; doi:10.1371/journal.pone.0162594)
Supplement: S1 Table — (DOCX) [file pone.0162594.s002.docx]

**S1. Table. FGFR2 Fusion Genes Identified in Intrahepatic Cholangiocarcinoma.**

| **Author** | **# samples with fusion/total iCCA samples (%)** | **FGFR2 fusions** |
| --- | --- | --- |
| Churi CR, et al. 2014^[29]^ | 3/55 (5) | FGFR2-KIAA1598, FGFR2-NOL4, FGFR2-PARK2 |
| Graham RP, et al. 2014^[5]^ | 12/96 (13) | Not provided |
| Sia D, et al. 2015 ^[27]^ | 48/107 (45) | FGFR2-PPHLN1, FGFR2-BICC1 |
| Borad MJ, et al. 2014^[20]^ | 3/6 (50) | FGFR2-BICC1, FGFR2-MGEA5, FGFR2-TACC3 |
| Arai Y, et al. 2014^[34]^ | 9/66 (14) | FGFR2-BICC1, FGFR2-AHCYL1 |
| Ross JS, et al. 2014^[30]^ | 3/28 (11) | FGFR2-BICC1, FGFR2-TACC3, FGFR2-KIAA1598 |
| Zheng Z, et al. 2014^[31]^ | - | FGFR2-CREB5 |
| Nakamura H, et al. 2015^[32]^ | 6/109 (6) | FGFR2-KCTD1, FGFR2-TXLNA, FGFR2-BICC1, FGFR2-AHCYL1 |
